# Users’ Perspectives, Opportunities, and Barriers of the Strengthen Your Ankle App for Evidence-Based Ankle Sprain Prevention: Mixed-Methods Process Evaluation for a Randomized Controlled Trial

**DOI:** 10.2196/rehab.8638

**Published:** 2018-07-06

**Authors:** Miriam van Reijen, Marianne Asscheman, Ingrid Vriend, Willem van Mechelen, Evert Verhagen

**Affiliations:** ^1^ Amsterdam Collaboration on Health and Safety in Sports Department of Public & Occupational Health, Amsterdam Public Health Research Institute VU University Medical Centre, Amsterdam Amsterdam Netherlands; ^2^ Consumer Safety Institute VeiligheidNL Amsterdam Netherlands; ^3^ School of Public Health, Physiotherapy and Population Sciences University College Dublin Dublin Ireland; ^4^ School of Human Movement and Nutrition Sciences Faculty of Health and Behavioural Sciences University of Queensland, Brisbane Brisbane Australia; ^5^ Division of Exercise Science and Sports Medicine Department of Human Biology, Faculty of Health Sciences University of Cape Town Cape Town South Africa

**Keywords:** injury prevention, ankle injury, eHealth, qualitative analysis, process evaluation

## Abstract

**Background:**

The “Strengthen Your Ankle” neuromuscular training program has been thoroughly studied over the past 8 years. This process evaluation is a part of a randomized controlled trial that examined both the short- and long-term effectiveness of this particular program. Although it was shown previously that the program, available both in a printed booklet and as a mobile app, is able to effectively reduce the number of recurrent ankle sprains, participants’ compliance with the program is an ongoing challenge.

**Objective:**

This process evaluation explored participants’ opinions regarding both the methods of delivery, using RE-AIM (Reach Effectiveness Adoption Implementation Maintenance) Framework to identify barriers and challenges to program compliance. Although Reach, Effectiveness, and Adaptation were the focus of a previous study, this paper focuses on the implementation and maintenance phases.

**Methods:**

Semistructured interviews and online questionnaires were analyzed using qualitative content analysis. Fisher exact, chi-square, and *t* tests assessed between-group differences in quantitative survey responses. Interviews were assessed by thematic analysis to identify key themes.

**Results:**

While there were no significant differences in the perceived simplicity, usefulness, and liking of the exercise during the 8 weeks of the neuromuscular training program, semistructured interviews showed that 14 of 16 participants agreed that an app would be of additional benefits over a booklet. After the 12-month follow-up, when asked how they evaluated the overall use of the app or the booklet, the users of the app gave a mean score of 7.7 (SD 0.99) versus a mean score 7.1 (SD 1.23) for the users of the booklet. This difference in mean score was significant (*P*=.006).

**Conclusions:**

Although both the app and booklet showed a high user satisfaction, the users of the app were significantly more satisfied. Semistructured questionnaires allowed users to address issues they would like to improve in future updates. Including a possibility for feedback and postponement of exercises, an explanation of the use of specific exercises and possibly music were identified as features that might further improve the contentment of the program, probably leading to increased compliance.

**Trial Registration:**

Netherlands Trial Register NTR4027; http://www.trialregister.nl/trialreg/admin/rctview.asp?TC=4027 (Archived by Webcite at http://www.webcitation.org/70MTo9dMV)

## Introduction

Injuries, due to participation in sports and physical activities, are prevalent. Internationally, ankle sprains are one of the most common musculoskeletal injuries [1]. In particular, indoor and court sports have shown high incidences of ankle sprains with up to 7 injuries per 1000 hours of participation [2]. Generally considered a “minor” injury, ankle sprains pose a significant risk for long-term secondary complaints like instability and chronic pain [3]. For the prevention of acute lateral ankle sprains, numerous effective strategies have been developed and evaluated for their cost-effectiveness [2].

One of the many available interventions that has been shown to be effective in reducing the risk of recurrent ankle sprains, as well as protecting against secondary complaints, is neuromuscular training (NMT) [3-5]. Multiple variations of such training programs have been evaluated [6-8], including the “Strengthen Your Ankle” program. The “Strengthen Your Ankle” program consists of 6 exercises that are performed 3 times a week, over 8 weeks. Multiple trials have indicated that this program can be effective in reducing the injury incidence density [9,10] as well as being cost-effective [10,11]. Despite the proven value of the program in preventing recurrent injury risk, compliance with this and other NMT programs is an ongoing challenge [3]. Sufficient compliance with NMT programs is essential for successful prevention of ankle sprains [12]. Consequently, a free mobile app was developed as a novel and attractive means of providing athletes with the “Strengthen Your Ankle” program [13]. Details of the app have been described elsewhere [3]. A recent trial (NTR 4027) showed that the app neither increased compliance nor decreased recurrence of ankle sprains compared with a standard program administered via a paper booklet [3,4,13].

As with other preventive interventions, the translation of the evidence on ankle sprain prevention through NMT to the real-world context of sports remains a challenge, by which effective ankle sprain prevention in the community is lagging [14]. The success of introducing any intervention strategy in a practical context can be evaluated using the RE-AIM framework [15]. RE-AIM is a conceptual framework that was originally used to develop and evaluate health care programs. The goal of the RE-AIM framework is to “encourage program planners, evaluators, readers of journal articles, funders, and policy makers to pay more attention to essential program elements, including external validity, that can improve the sustainable adoption and implementation of effective, generalizable, evidence-based interventions” [16].

Although developed for use in health care settings, the RE-AIM framework has been previously used to evaluate the success of introducing strategies for sports injury prevention within a practical sports context [17,18]. Consequently, using the components of the RE-AIM framework, this study described the user experience of the “Strengthen Your Ankle” app and booklet to understand why compliance was challenged during program implementation.

## Methods

### Design and Participants

The full details of the “Strengthen Your Ankle” study have been described elsewhere [3,4,13]. In brief, 220 sports participants who experienced an ankle sprain during the past 2 months were included in this RCT. Participants were randomly assigned to either the app or booklet intervention group and were instructed to follow the embedded 8 week “Strengthen Your Ankle” NMT prevention program using either the app or the printed booklet.

### Outcome Measures

The RE-AIM framework describes five dimensions to evaluate the practical feasibility of an intervention: “Reach,” “Effectiveness,” “Adoption,” “Implementation,” and “Maintenance” [16]. The dimensions “Reach” and “Adoption” are out of scope when describing the feasibility of an intervention within a controlled trial. As such, for the this study, we focused on the dimensions “Effectiveness,” “Implementation,” and “Maintenance.”

### Effectiveness

The “Effectiveness” dimension describes the clinical impact of the studied intervention. The short- and long-term effectiveness of the app compared with the booklet for preventing ankle sprain recurrences were assessed in a RCT. The full methods and results of this trial have been published elsewhere [3,4,13]. In order to put the outcomes of the “Implementation” and “Maintenance” dimensions in context, we will briefly summarize the “Effectiveness” outcomes.

### Implementation

Implementation concerns the participants’ use of the intervention strategies. In this study, we quantified use as compliance with the 8-week NMT program in each of the study groups, measured as a percentage of the total program completed. In addition, the participants’ attitudes and perceptions toward the delivery of the NMT programs were assessed.

During the 8 weeks of the NMT program, participants received a weekly online questionnaire. The questionnaire registered what percentage of the program was executed during the week, the amount of difficulty that was experienced while conducting each of the exercises, and the reason for a possible lack of compliance. For each of the 6 different exercises, participants indicated what percentage of the exercises they performed each week. Additionally, using a 5-point Likert scale, participants were asked how they perceived the exercises. When participants failed to complete the questionnaire, reminders were sent by email. The details on the questionnaire have been published previously [3].

After the 8-week training period, a more extensive evaluation questionnaire was completed, including closed and free-text questions on the subjectively-experienced value of the NMT program delivery mode, a subjective evaluation of the program, and the perceived disadvantages and advantages of the allocated intervention delivery mode. To measure satisfaction, all remaining participants (75 in the app group and 88 in the booklet group) were asked to give a 0-10 score for the app or booklet. An unpaired *t* test was performed to examine the difference in scores between the two groups.

### Maintenance

“Maintenance” describes the long-term effectiveness of the intervention strategies. For this study, this dimension was defined as the percentage of participants still conducting the NMT program combined with the advantages the participants perceived related to the app or paper booklet use for intervention delivery.

After 12 months, semistructured interviews were conducted with individual participants to assess the perceived advantages of using the app over the paper booklet. All study participants were asked if they were willing to participate in a semistructured interview concerning the NMT program; 27% (32/119) of the remaining participants, evenly divided over the two study groups, responded positively. The interviews were structured using a preselected topic list on the individual experiences with the NMT program either through the booklet or app. All interviews were conducted and transcribed by one researcher (MA). Interviews were conducted via telephone until saturation was reached, that is, when interviews did not lead to new themes or information, within both study groups, resulting in 16 semistructured interviews with 8 randomly selected participants in the booklet group and 8 randomly selected participants in the app group. Multimedia Appendix 1 shows the question guide for the semistructured interviews, aimed at process evaluation, after finishing the 12-month intervention.

### Data Analyses

Due to dropout during follow-up (n=57 after 8 weeks and a further n=44 after 12 months), sample sizes differed between questionnaires. The reasons for dropout were unknown. The participants’ answers on the 5-point Likert scales regarding attitudes and perceptions toward the program, as registered during the 8-week program, were averaged for each participant over the available follow-up moments. Independent sample *t* tests with assumed equal variances were conducted to assess for differences in the average Likert responses between the two study groups. The significance level was evaluated at *P*=.05. SPSS (version 22.0) and was used for all statistical analyses.

All semistructured interviews were audiorecorded and transcribed verbatim. In transcriptions, any personal information or information that was deducible to an individual was anonymized. Verbatim-transcribed interviews were thematically analyzed and fragmented on the basis of topical similarity using Atlas.ti [19]. Open, inductive coding was used line by line on the transcripts of the interviews and these codes were converged into subthemes [20]. Peer debriefing was used as an external check to the research process. This method of analysis was used after each interview and ended when no new codes arose and saturation was reached [19]. The final step in the analysis process was to submerge the subthemes to a limited number of main themes [19].

## Results

### Effectiveness

Previous studies that looked at the effectiveness of the “Strengthen Your Ankle” program provided further details on the (cost)-effectiveness of the program in the short and long term [3,4]. In short, during the 8 weeks of the NMT, there were 93 self-reported recurrent ankle sprains, which resulted in injury incidence densities of 25.3 per 1000 hours of sport (95% CI 18.0 to 32.7) in the app group and 25.6 per 1000 hours of sport (95% CI 18.3 to 32.9) in the booklet group. There was no significant difference in the incidence densities of self-reported recurrences (HR [hazard ratio] 3.07; 95% CI 0.62 to 15.20) [1].

During the 12-month follow-up, there were 139 recurrent ankle injuries, resulting in injury incidence densities of 15.59 per 1000 hours of sport (95% CI 11.94 to 19.24) in the app group and 15.84 (95% 12.10 to 19.58) in the booklet group. Over the long term, this difference in injury density was not significant (HR 1.06; 0.76 to 1.49) [4].

### Implementation

The first study in this larger research project looked at compliance during the 8 weeks of the NMT intervention. It was shown that the average compliance to the exercise scheme was 73.3% (95% CI 67.7% to 78.1%) in the app group and 76.7% (95% CI 71.9 to 82.3%) in the booklet group. No significant difference in compliance was found between the groups [3].

The weekly questionnaires (Table 1) showed that participants in both the app and booklet groups gave comparable scores with regard to simplicity, usefulness, and subjective evaluation of the exercises. Table 1 shows the averaged responses of the participants over the 8 weeks.

After the 8-week intervention period, 35 participants using the app and 22 participants using the booklet discontinued the study for unknown reasons. The remaining 75 users of the app found this method of NMT program delivery more user friendly, easier, fun to use, and less annoying and thought that the videos were more helpful than the booklet (Table 2). The latter question should be interpreted with caution because online videos were available for the booklet users (n=88), but many of the participants stated that they were not aware of this possibility.

**Table 1:** Participants’ attitudes and perceptions toward the allocated delivery of the NMT program during the 8-week intervention period.

Participants’ opinions and method of delivery		Mean (SD)^a^	Mean difference^b^ (95% CI)	*P* value
**The exercises are simple.**			0.03 (−0.19 to 0.25)	.79
	App	3.79 (0.86)		
	Booklet	3.76 (0.78)		
**Due to the variation in exercises I stay motivated.**			−0.16 (−0.36 to 0.05)	.13
	App	2.25 (0.82)		
	Booklet	2.41 (0.71)		
**I find it easy to execute the exercises without help.**			0.05 (−0.16 to 0.26)	.65
	App	3.72 (0.85)		
	Booklet	3.67 (0.75)		
**The exercises give me a sense of security.**			−0.01 (−0.25 to 0.23)	.96
	App	3.30 (0.94)		
	Booklet	3.30 (0.87)		
**The exercises are painful.**			−0.04 (−0.22 to 0.14)	.64
	App	3.94 (0.68)		
	Booklet	3.98 (0.67)		
**The exercises don’t fit with my regular schedule.**			0.09 (−0.14 to 0.32)	.47
	App	3.42 (0.87)		
	Booklet	3.33 (0.88)		
**I have too little time to do the exercises.**			−0.09 (−0.35 to 0.17)	.49
	App	3.29 (0.99)		
	Booklet	3.38 (0.97)		
**I think the exercises take a long time.**			−0.15 (−0.32 to −0.01)	.07
	App	2.00 (0.58)		
	Booklet	2.16 (0.67)		
**The exercises make me tired.**			−0.02 (−0.21 to 0.17)	.84
	App	3.87 (0.75)		
	Booklet	3.89 (0.66)		
**I forget to execute the exercises.**			−0.06 (−0.24 to 0.11)	.49
	App	2.34 (0.68)		
	Booklet	2.41 (0.64)		
**The exercises are not useful to prevent a recurrent injury.**				
	App	3.42 (0.88)	0.12 (−0.11 to 0.35)	.32
	Booklet	3.31 (0.84)		
**The exercises won’t help me.**			0.07 (−0.13 to 0.26)	.50
	App	2.66 (0.77)		
	Booklet	2.59 (0.71)		

^a^Scores present means (SD) of 5-point Likert scales (1=strongly agree; 5=strongly disagree).

^b^Differences in scores between groups were analyzed by independent *t* tests with equal variances assumed.

**Table 2:** The subjectively-experienced value of the NMT program and perceived disadvantages and advantages of the allocated intervention delivery mode assessed directly after the 8-week intervention.

Participants’ opinions and method of delivery		Mean (SD)^a^	Mean difference^b^ (95% CI)	*P* value
**The intervention is user friendly.**			−0.43 (−0.75 to −0.11)	.009
	App	1.85 (0.98)		
	Booklet	2.28 (1.10)		
**The intervention is easy to use.**			−0.40 (−0.69 to −0.11)	.008
	App	1.84 (0.92)		
	Booklet	2.24 (0.97)		
**The intervention looks attractive.**			−0.06 (−0.35 to 0.23)	.68
	App	2.12 (0.90)		
	Booklet	2.18 (0.97		
**Navigation of the intervention is clear.**			−0.29 (−0.59 to 0.01)	.06
	App	2.13 (0.95)		
	Booklet	2.42 (1.01)		
**The intervention gives enough information.**			−0.29 (−0.59 to 0.01)	.06
	App	2.19 (0.95)		
	Booklet	2.48 (0.97)		
**I would advise others to use the intervention.**			−0.29 (−0.62 to 0.03)	.07
	App	2.08 (1.03)		
	Booklet	2.38 (1.04)		
**It is annoying to use the intervention.**			0.47 (0.12 to 0.81)	.008
	App	4.09 (1.09)		
	Booklet	3.63 (1.13)		
**I have used the intervention with pleasure.**			−0.18 (−0.48 to 0.12)	.23
	App	2.25 (0.95)		
	Booklet	2.44 (0.97)		
**The videos helped me (online for the Booklet).**			−0.99 (−1.31 to −0.68)	< .001
	App	1.96 (1.07)		
	Booklet	2.95 (0.96)		
**The written instructions helped me.**			−0.07 (−0.35 to 0.21)	.64
	App	2.08 (0.98)		
	Booklet	2.15 (0.84)		
**The schedule helped me.**			0.08 (−0.23 to 0.38)	.62
	App	2.12 (1.10)		
	Booklet	2.05 (0.87)		
**The intervention is boring.**			−0.05 (−0.36 to 0.26)	.73
	App	3.48 (1.03)		
	Booklet	3.53 (0.97)		
**The intervention makes it easier to do the exercises.**			−0.36 (−0.65 to −0.07)	.02
	App	2.09 (0.94)		
	Booklet	2.45 (0.95)		
**The intervention makes it fun to do the exercises.**			−0.37 (−0.66 to −0.08)	.01
	App	2.68 (0.94)		
	Booklet	3.06 (0.93)		
**The intervention is informative.**			−0.14 (−0.39 to 0.11)	0.26
	App	2.20 (0.74)		
	Booklet	2.34 (0.84)		
**The intervention is trustworthy.**			−0.17 (−0.42 to 0.09)	0.13
	App	2.23 (0.84)		
	Booklet	2.40 (0.870)		
**The explanation of the exercises is clear.**			−0.22 (−0.52 to 0.10)	0.17
	App	2.26 (1.07)		
	Booklet	2.47 (0.91)		

^a^Scores present means (SD) of 5-point Likert scales (1=strongly agree; 5=strongly disagree).

^b^Differences in scores between groups were analyzed through independent *t* tests with equal variances assumed.

Therefore, the answers of 53 of the booklet users were “neutral” when asked if the online videos were of help; this was in comparison with 5% (4/75) in the app group. Some participants failed to answer all the questions, the number of missing responses can be found in Table 2. Additional questions specifically related to possible improvements in the app, and not the booklet, (Multimedia Appendix 2) indicated that participants desired feedback after the exercises (44/75, 59%) and wanted the ability to postpone a training session (41/75, 55%). Overall, a *t* test showed that the users of the app were significantly more satisfied with the app (score 1 out of 10 with 10 referring to the highest score, mean±SD) compared with booklet users; 7.7 (SD 0.99) versus 7.1 (SD 1.23) *P=*.006.

### Maintenance

At the end of the 12-month follow-up period, an additional 44 participants discontinued the study. These participants were asked if they were still doing (part of the) NMT program. Only 23% (28/122) of all participants still in the study responded affirmatively. We did not ask what amount of the program they were still doing.

Two main themes arose from the semistructured interviews that related to the design of the app and possible additional benefits of the app. Fourteen out of 16 participants stated that an app would provide an additional benefit compared with a booklet. The main reasons given were that most of the participants always had their mobile phones with them and that the app provided visual support and had a reminder function. The two participants who did not feel that the app offered any benefit found the exercises too easy, which made the app redundant.

Errors in navigation and explanation, the lack of feedback and music, and lack of explanation of the purpose of the exercises were the main disadvantages experienced by the app users. The greatest perceived disadvantages of the booklet were the big size when folded out, small font, lack of robustness, and errors in explanation. Table 3 shows the individual responses during the semistructured interviews to illustrate the flavor of the original data and demonstrate the prevalence of the themes, as suggested by King [21].

**Table 3:** Individual responses from semistructured interviews.

Method of delivery and respondent		Added benefit of the app?	Reason given	Pros (+) and suggestions for improvement (−) for the app
**App**				
	R1	Yes	You always have your phone with you You forget the booklet	+ Easy to use + Agenda function + Videos with instructions
	R2	No	The exercises are so easy, you don’t need an app	+ Videos with instructions + Tick off done exercises
	R3	Yes	You always have your phone with you Seeing the app on my phone reminds you to do the exercises	+ Tick off done exercises − Show why you need to do an exercise
	R4	Yes	The app gives visual support	+ Easy to use + Videos with instructions
	R5	Yes	You always have your phone with you	+ Easy to use
	R6	Yes	The app is smaller and thus easier to use	+ Easier navigation
	R7	Yes	The app gives visual support Seeing the app on my phone motivates you to do the exercises	+ Videos with instructions + Counting down the number of exercises
	R8	Yes	You always have your phone with you	+ Videos with instructions + Tick off done exercises
**Booklet**				
	R9	Yes	You always have your phone with you	− Show why you need to do an exercise. + Reminder to do the exercises.
	R10	No	The exercises are so easy, you don’t need an app	− Stopwatch function
	R11	Yes	The app gives visual support	− Show why you need to do an exercise
	R12	Yes	You always have your phone with you	+ Reminder to do the exercise − Possibility to postpone exercises
	R13	Yes	The app gives visual support	+ Videos with instructions
	R14	Yes	You always have your phone with you Seeing the app on my phone would remind you to do the exercises	− Direct translation of the app to a booklet
	R15	Yes	You always have your phone with you	− More variation in the exercises
	R16	Yes	An agenda function would be easy	− Direct translation of the app to a booklet

## Discussion

### Principal Findings

Previous studies [3,4] have shown that using an app or a booklet with a special NMT program to prevent recurrent ankle sprains has resulted in comparable injury densities during both short- (8 weeks) and long-term (12 months) follow-ups and comparable compliance rates with the program. During the execution of the program during the first 8 weeks, the app and booklet were given comparable scores for simplicity, usefulness, and liking of the exercises. After the 12-month follow-up, the users of the app were significantly more satisfied with the app compared with the users of the booklet. The users of the app evaluated the app as more patient friendly, easier to use, and less annoying and thought that the videos were helpful. With the help of semistructured interviews, 14 out of 16 participants agreed that an app would be of additional benefit over a booklet, mainly due to use of instructional videos, phone portability, and the agenda function. Further suggestions for improving the app that were mentioned by various participants were the ability to postpone exercises and the provision of exercise feedback.

Interventions for preventing sport injuries require high participant compliance [3] Therefore, ways to increase compliance are a focus of many intervention studies [3]. The “Strengthen Your Ankle” program was developed in 2009. Since then, the program has been studied intensively [3,4,9-11,22]. It was shown that (1) the program was effective in reducing recurrent ankle sprains for those with high compliance [10], (2) the use of either the app or a booklet produced nonsignificant differences in injury densities in both the short and long term [3,4], and (3) both methods had comparable cost-effectiveness of implementation [23].

Over the years, compliance with the “Strengthen Your Ankle” program in RCTs has steadily increased from 23% [9] to 45% [10] and 75% [3], likely as a result of annual updates, increased acknowledgment of the usefulness of the program by the target population, and improvements in the program content. However, the reach of the target population still requires substantial attention. In 2011, the annual number of downloads of the “Strengthen Your Ankle” app reached 25,781, which corresponds to a low percentage (25,781/911,576, 2.6%) of potential users [18]. Some studies have looked at the use of apps in injury prevention over the last decade. What can be concluded from those studies is that numerous apps seek to prevent (re)injury. However, the scientific evidence supporting these app-based programs is nonexistent or scarce [22,24]. A recent review found that out of 18 apps concerned with preventing sports and physical activity-related injuries, only four included evidence regarding efficacy [22]. In addition to the app that is the focus of this study, one of those four apps dealt with ankle injury prevention using NMT. No information is available on the use or compliance of the other app [22].

This study aimed to explore user experiences with the NMT program, as well as with the app and booklet as delivery methods, by means of semistructured interviews. The information gathered can be used to further improve the methods of delivery and, thus, increase future reach and compliance. The interviews and questionnaires showed that the app and booklet can be successfully used to prevent recurrent ankle sprains and that both show high user satisfaction. Future updates may include options for feedback or postponement of exercises, an explanation of the use of specific exercises, and possibly music; these additions could further improve user perceptions of the program and hence increase compliance.

A limitation of this study, and that of previous studies on the “Strengthen Your Ankle” program, is the mismatch between compliance and adherence. Although both constructs have been used interchangeably, they are not synonymous. Adherence refers to a situation where a clinician or researcher develops a program in cooperation with the participant. The participant attempts to follow the program as best as possible, taking personal preferences and constraints into consideration. Adherence can be seen as what happens in real-life conditions when individuals with an ankle sprain try to follow the program; compliance is studied in clinical settings. The extent to which the participant obeys the program instructions is measured by compliance rates [12,24]. Research, ideally performed in a more or less controlled setting, implicitly focuses on compliance, rather than on adherence. However, the “Strengthen Your Ankle” program is meant to increase adherence for all individuals at risk for an ankle sprain, not only for those who participate in the studies involved. This study has tried to explore the barriers and opportunities that participants experienced while using the training program via an app or booklet within a controlled study setting. However, because the interviews were held after follow-up, that is, months after the participants had finished the 8 weeks of the training program, we expected to gain insight as to program performance in real-life situations.

A further limitation of this study is the possibility of selection bias for the semistructured interviews. It is possible that only those participants that carried a strong negative or positive view of the program agreed to participate because the invitation for the interviews was made only after termination of the 12-month follow-up. Additionally, the (single) interviewer did not structure the interviews and continued to question the participants when needed. This may have affected the validity of the data analyses. However, it is recognized that this characteristic is inherent to the flexible nature of thematic analysis and does not threaten the depth of analysis [5].

### Conclusions

With the use of semistructured interviews and online questionnaires, we were able to evaluate users’ opinions on both the app and booklet. The users of the app were significantly more satisfied with the app although there was no significant difference in the perceived simplicity, usefulness, and liking of the exercise during the 8 weeks of the NMT program. In the interviews, users acknowledged the need for improvements. Future updates should take the users’ suggestions into account because adherence with the NMT program remains an ongoing challenge.

## Appendix

Multimedia Appendix 1 Question guide for the process evaluation using semi-structured interviews after finishing the 12-month intervention period.

Multimedia Appendix 2 Responses to process evaluation of the neuromuscular training program after the intervention period.

## Abbreviations

ESSM: exercise science and sports medicine
HR: hazard ratio
NMT: neuromuscular training
RCT: randomized controlled trial
ZKA: Zilveren Kruis Achmea
