# Supplementary material for: Users’ Perspectives, Opportunities, and Barriers of the Strengthen Your Ankle App for Evidence-Based Ankle Sprain Prevention: Mixed-Methods Process Evaluation for a Randomized Controlled Trial
Source: JMIR Rehabil Assist Technol. 2018 Jul 6;5(2):e13. doi: 10.2196/rehab.8638 (PMC6054707; doi:10.2196/rehab.8638)
Supplement: Multimedia Appendix 1 [file rehab_v5i2e13_app1.pdf]

Multimedia Appendix 1: Question guide for the process evaluation using semi-structured interviews after finishing the 12-month intervention period.

**Question guide for the App**
**It is true that you have used the App to follow the Strengthen Your Ankle training program?**
**Did you execute the 8 weeks of the training program, as instructed?**<br>**If no, what was the reason for not following the training program as instructed?**<br>**How much of the training program did you follow?**<br>**Do you still follow the training program now?**
**Can you elaborate on how you have experienced:**<br>- **The usability**<br>- **The provided information**<br>- **The design**
**How did the app contribute to the Strengthen Your Ankle program?**
**If the App were updated, what would you like to improve?**
**Currently there are many medical applications available.**<br>**Would you only use these apps when a medical professional advice you to do so?**
**Would you advise others to use the App to follow the Strengthen Your Ankle training program?**
**Are there any remarks you want to make concerning the App or the program?**

**Question guide for the Booklet**
**It is true that you have used the booklet to follow the Strengthen Your Ankle training program?**

**Did you execute the 8 weeks of the training program, as instructed?**<br>**If no, what was the reason for not following the training program as instructed?**<br>**How much of the training program did you follow?**<br>**Do you still follow the training program now?**
**Can you elaborate on how you have experienced:**<br>- **The usability**<br>- **The provided information**<br>- **The design**
**You might be aware of the fact that there is also a mobile App available with the Strengthen Your Ankle program. Do you think the app would contribute to the training program?**<br>**And if yes, how?**
**How would you like a mobile application to be developed?**<br>**What features would you prefer?**
**Currently there are many medical applications available.**<br>**Would you only use these apps when a medical professional advice you to do so?**
**Would you advise others to use the Strengthen Your Ankle training program?**
**Are there any remarks you want to make concerning the possibility of an App or the program?**
