# Supplementary material for: Users’ Perspectives, Opportunities, and Barriers of the Strengthen Your Ankle App for Evidence-Based Ankle Sprain Prevention: Mixed-Methods Process Evaluation for a Randomized Controlled Trial
Source: JMIR Rehabil Assist Technol. 2018 Jul 6;5(2):e13. doi: 10.2196/rehab.8638 (PMC6054707; doi:10.2196/rehab.8638)
Supplement: Multimedia Appendix 2 [file rehab_v5i2e13_app2.pdf]

MULTIMEDIA APPENDIX 2: Responses to process evaluation of the neuromuscular training program after the intervention period

	What are the three greatest benefits of the App?	What are the three greatest disadvantages of the App?	Verbatim examples
**Visual aspects**	Video's and sounds<br>Easy to navigate<br>Looks good	Errors in navigation<br>Need to have phone at hand<br>No possibility to postpone	"The videos help me to do the exercises because they show me how I should do them"
**Informational aspects**	Schedule<br>Information on brace/tape<br>Written information	Errors in explanation<br>Lack of explanation of purpose of exercise	"The app helped me with information on the exercises"
**Motivational aspects**	The reminder function<br>Telephone is always at hand<br>Less boring to do the exercises	Lack of feedback<br>No timer function<br>No music	"I would love to get feedback on why I should do a particular exercise and what exactly I'm training"
	What are the	What are the	

	three greatest benefits of the Booklet	three greatest disadvantages of the Booklet?	
**Visual aspects**	Compact<br>Looks good<br>Strong material	No possibility to turn pages<br>Big when folded out<br>Small letters<br>Lack of videos<br>Not robust<br>Not easy to unfold	“If I knew how the exercises looked on a video this might help me to do them correctly”
**Informational aspects**	Schedule<br>Figures<br>To the point<br>Information on brace/tape	Too short in information<br>Difficult schedule<br>Errors in explanation	“The schedule was very clear and showed me what I should do at what moment”
**Motivational aspects**	Easy to carry	Lack of feedback	“I would like to see a picture of what muscles I’m training and get feedback on how I do the exercises”
